# Intra-articular Localized Haemangioma of the Knee Mimicking Localized Pigmented Villonodular Synovitis: A Case Report

**DOI:** 10.5704/MOJ.1703.004

**Published:** 2017-03

**Authors:** GK Goki-Kamei, NM Norimasa-Matsubara, TT Teruyasu-Tanaka, KN Koji-Natsu, TS Toshihiro-Sugioka

**Affiliations:** Department of Orthopaedics, Miyoshi Central Hospital, Miyoshi, Japan

**Keywords:** synovial haemangioma, pigmented villonodular synovitis, arthroscopic resection

## Abstract

Intra-articular synovial haemangioma of the knee is a benign tumour. However, diagnostic delay leads to degenerative changes in the cartilage and osteoarthritis due to recurrent haemarthrosis. Therefore, treatment should be performed immediately. We report the case of a localized synovial haemangioma arising from the medial plica in a 38-year old female presenting with pain and restricted range of motion in the right knee joint. Initially, we diagnosed this case as a localized pigmented villonodular synovitis (LPVS) based on MRI and arthroscopic findings and performed only arthroscopic en bloc excision of the mass and synovectomy around the mass for diagnostic confirmation. Fortunately, there was no difference in the treatment approaches for LPVS and localized haemangioma and the synovial haemangioma had not recurred at the 3-month postoperative follow-up with MRI. The patient’s clinical symptoms resolved and had not relapsed two years after surgery.

## Introduction

Synovial haemangioma of the knee is a rare benign tumour first described by Bouchut in 1856. Approximately 200 cases have been reported since then^1^. The usual clinical symptoms of synovial hemangioma include swelling, pain, limitation of range of motion (ROM), and recurrent haemarthrosis, especially in the absence of specific trauma. They are most commonly seen during childhood and early adulthood. However, this condition does not have specific symptoms; thus, delay in the diagnosis is a major issue. In particular, immediate diagnosis and treatment are expected because such a delay can leads to degenerative changes in the cartilage and osteoarthritis in case of recurrent haemarthrosis.

We report the case of a localized synovial hemangioma arising from the medial plica in a 38-year old female presenting with pain and ROM restriction in the right knee joint; we made this diagnosis at two weeks after the onset of symptoms.

## Case Report

**Fig. 1:**
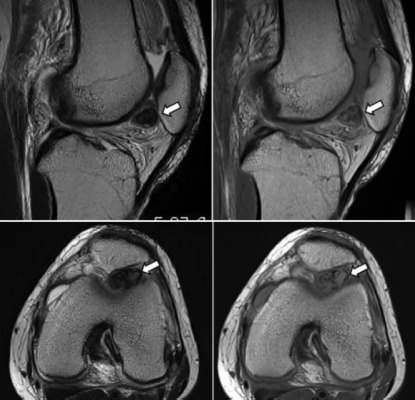
The MRI revealed a solitary mass in the knee joint cavity or infrapatellar fat pad that was hypointense-to-isointense in proton-density-weighted images and isointence-to-hyper-intense in T2-weighted images.

A 38-year old female presented with right knee pain without an obvious cause. The ROM in the right knee was limited and she had right knee pain while walking. Because her symptoms persisted for a week, she consulted our hospital. At her initial visit, there was swelling, effusion, ROM limitation (from 30 degree extension to 90 degree flexion), and marked tenderness of the patella-femoral joint on physical examination. She had severe knee pain on walking and twisting the knee. Aspiration of the right knee joint yielded yellow serous fluid. However, her symptoms were not relieved. Therefore, we suspected the presence of a loose body, discoid lateral meniscus, or intraarticular tumor and carried out a magnetic resonance imaging (MRI) scan. Pigmented villonodular synovitis (PVS) or ganglion was suspected based on the MRI findings (Fig. 1). The patient was provided with detailed information about characteristic of the tumour and surgical procedures (arthroscopic excision or open excision). We also obtained informed consent for publication.

**Fig. 2:**
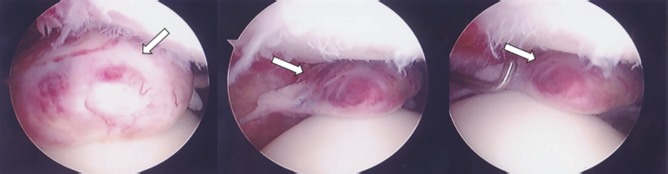
Arthroscopic findings. The solitary mass (approximately 20 × 20 × 10 mm) around the medial patello-femoral joint and originating from the plica or the capsule.

**Fig. 3:**
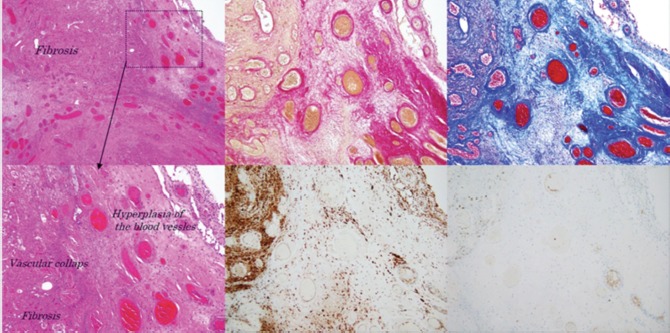
HE staining showing fibrosis, necrotic tissue and hemosiderin-laden macrophages in the interstitial tissue surrounding the blood vessel cavities. The outer layer of the mass was wrapped in synovium. Immunohistological staining showed hyperplasia of the capillaries and arteriolovenous vessels (Elastica van Gieson and Factor 8 stains), a capsule coated with relatively thick collagen fibers (Masson trichrome stain) and accumulation of hemosiderin in the synovium (CD68). (Upper: left; HE×40 (magnification), middle; EVG×100, right; Masson trichromex100, Bottom: left; HE×100, middle; CD68×100, right; Factor 8×100)

An arthroscopy was performed on the right knee joint to view the status of the solitary mass via an infra-patellar incision. We performed en bloc excision of the mass, with arthroscopic synovectomy of the tissue around the mass (Fig. 2). The histological findings and the diagnosis corresponded to a synovial haemangioma (Fig. 3) The appearance of the surrounding synovial tissue was normal.

Her symptoms, including severe tenderness of the patello-femoral joint and ROM limitation disappeared soon after the surgery. The clinical outcome was assessed using the Knee Osteoarthritis Outcome Score (KOOS) that includes five subgroups: symptom, pain, activities of daily living (ADL), quality of life (QOL), and sports. Each subgroup of the KOOS score dramatically improved three months postoperatively: symptom score (10.71 to 71.43), pain score (41.67 to 97.22), ADL score (23.53 to 98.53), QOL score (0 to 100), and sports (0 to 100).The synovial haemangioma had not recurred at the 3-month postoperative follow-up with MRI.

## Discussion

Differential diagnoses of synovial haemangioma in the knee joint include synovial chondromatosis, PVS, ganglion, giant cell tumor, synovial sarcoma, and lipoma among other conditions. Although we can usually distinguish the tumour by the clinical symptoms and the arthroscopic and MRI findings, definitive diagnosis is based on the histological findings. In the present case, the MRI and arthroscopic findings made us initially suspect localized PVS (LPVS). Additionally, this patient was older than the mean age of onset of synovial haemangioma previously reported ^2^. Therefore, we performed only arthroscopic en bloc excision of the mass and synovectomy around the mass to confirm the diagnosis, because the recurrence of LPVS has not been reported. However, the definitive diagnosis by the histologic findings was synovial haemangioma. We did not keep in mind synovial haemangioma because intra-articular synovial haemangioma of the knee was a rare benign tumour and the localized type was very rare especially among this tumour type. Fortunately, there was no difference in the treatment approaches for LPVS and localized synovial haemangioma. Therefore, the treatment could be successfully completed without any complications.

The most serious issue related to intra-articular hemangioma is the degeneration of the articular cartilage due to recurrent haemarthrosis. In particular, diagnostic delay leads to degenerative changes in the cartilage and osteoarthritis. Therefore, immediate diagnosis and treatment should be performed. Secondly, the tumour could spread throughout the synovium and gradually infiltrate the surrounding muscles. Treatment options for similar cases include open surgical resection, arthroscopic excision, arthroscopic ablation and embolization, and the treatment policy is generally decided by the classification type. Bennet and Cobey classified synovial haemangiomas as either localized (pedunculated) or diffuse type^3^. The localized type can be completely resected arthroscopically and has a good prognosis^2^. However, it is difficult for the diffuse type to be completely resected arthroscopically, and substantial bleeding is a concern. Therefore, open excision with synovectomy is the most widely used approach for the management of the diffuse type. Although the recurrence rate of the synovial haemangioma was unclear, the local recurrence rate was high in cases with cartilage degeneration^4^. There were several other reports regarding recurrence after the resection of synovial haemangiomas (diffuse type) in infants^5^. There has been no reports of the recurrence of localized synovial haemangioma.

Regardless, accurate diagnosis and early appropriate treatment are necessary for synovial haemangiomas as for other tumours. In the present case, the synovial haemangioma had not recurred at the 3-month postoperative follow-up with MRI. The patient’s clinical symptoms resolved and had not relapsed at two year after the surgery. Careful observation and continuous monitoring are needed because the recurrence of intra-articular haemangiomas has been previously reported.

There is no conflict of interest in this report. We thank Prof. H Kuniyasu (Nara Medical University) for his assistance (for Fig. 3).
